# Surface Calcium Enrichment of Biogenic Hydroxyapatite Regulates Macrophage Inflammation via Toll-Like Receptor 4 Signaling

**DOI:** 10.34133/bmr.0409

**Published:** 2026-09-01

**Authors:** Meihua Mai, Mengxi Su, Shudan Deng, Tianze Lin, Junming Feng, Chunhsin Hsu, Chuangji Li, Zefeng Guo, Xinyi Yang, Shiyu Wu, Zhuofan Chen

**Affiliations:** ^1^ Hospital of Stomatology, Guanghua School of Stomatology, Sun Yat-sen University and Guangdong Provincial Key Laboratory of Stomatology, Guangzhou 510055, China.; ^2^ Zhuhai Hospital of Integrated Traditional Chinese and Western Medicine, Zhuhai, China.

## Abstract

Biogenic hydroxyapatite (BHA) is widely used for oral bone defect regeneration, yet its osteogenic efficacy is limited by slow osteogenesis and insufficient bone volume, which may be attributed to early inflammatory responses. The investigation of granular biomaterials is further challenged by conventional in vitro models that fail to ensure adequate cell–material interactions and efficient RNA extraction, limiting experimental reliability. Here, we developed an improved direct coculture system together with an optimized RNA extraction strategy to address these limitations. Using this platform, we characterized BHA-induced inflammatory responses at the gene, protein, and transcriptomic levels. Our results showed that the inflammatory activity of BHA is associated with its calcium-enriched surface, which activates the Toll-like receptor 4 signaling pathway. Furthermore, a calcium pre-adsorption strategy was introduced to modulate surface ion activity, which reduced calcium enrichment and attenuated inflammatory signaling. These findings suggest that modulating calcium enrichment capacity may offer a promising strategy to regulate the immune response of hydroxyapatite-based bone substitutes and improve their biological performance.

## Introduction

Oral bone defects caused by periodontitis, trauma, or tumor resection have led to a growing demand for bone grafts and biomaterials [1–3]. Among available options, biogenic hydroxyapatite (BHA) is widely used in clinical practice for guided bone regeneration (GBR) and alveolar ridge preservation (ARP), due to its good osteoconductivity as a bioactive scaffold that enables the ingrowth of osteoblasts [4,5]. As a xenogeneic bone substitute, BHA eliminates the need for a second surgical site compared to autogenous bone. However, its osteogenic efficacy remains limited by slow bone formation and compromised bone volume [6,7], which may be partly attributable to early inflammatory responses induced by the material.

The early inflammatory responses following biomaterial implantation play a critical role in regulating subsequent tissue regeneration [8,9], where an appropriate inflammatory level is beneficial, whereas excessive or prolonged inflammation impairs bone formation [10,11]. Therefore, elucidating material-induced inflammatory effects and their underlying mechanisms, as well as developing immunomodulatory strategies, is essential for improving osteogenic outcomes. Recent studies have reported an overactivated pro-inflammatory immune microenvironment during BHA-mediated healing in vivo [12,13]. However, in vivo inflammatory responses are influenced by multiple confounding factors, including biomaterial-mediated effects, soft tissue thickness, surgical procedures, and prolonged surgical duration [14–16], making it difficult to isolate the intrinsic immunological effects of the material itself. Therefore, a controlled in vitro model is required to specifically evaluate the material-induced inflammatory responses.

Current in vitro models for studying materials mainly include indirect and direct culture approaches [17]. In the indirect model, materials are incubated in culture medium, and the resulting supernatant is collected as the “Extract”. This approach is widely employed to assess the effects of ions or soluble components released from the material [13,18]. Since cell adhesion to the material surface is the initial event that profoundly influences subsequent cellular responses [19,20], the indirect model does not permit cell–material contact and has inherent limitations.

In contrast, in the direct culture model, materials are added directly into the culture system, allowing physical interaction between cells and materials [21,22]. However, when granular materials are used, especially at the millimeter scale, they tend to disperse unevenly in the medium, resulting in incomplete and inconsistent cell–material contact. In addition, RNA extraction in such systems remains technically challenging, as negatively charged RNA is adsorbed to the material surface during cell lysis, leading to reduced RNA yield and quality [23]. Collectively, these limitations highlight the lack of a reliable system capable of ensuring consistent cell–material interactions and efficient RNA extraction.

To address these limitations, we developed an improved direct coculture system to ensure uniform and reproducible cell–material interactions. In addition, a “pre-separation” strategy was introduced for RNA extraction to minimize material-associated RNA loss and improve RNA quality. Based on this model, we systematically investigated the inflammatory signaling pathways activated by BHA and further explored the regulatory role of surface calcium enrichment through a calcium pre-adsorption strategy. Our findings reveal the relationship between surface physicochemical properties and inflammatory responses, providing a mechanistic basis for the rational design of immunomodulatory bone substitutes.

## Materials and Methods

### BHA preparation

BHA was derived from porcine cancellous bone according to a standardized protocol as previously described [24–26]. The hydroxyapatite particles were ground to a size range of 0.25 to 1 mm and sterilized prior to use.

### Calcium enrichment activity evaluation

To analyze the calcium enrichment activity of BHA, the BHA was incubated in complete medium at a ratio of 180 mg/ml at 37 °C for 24 h. Supernatant was collected by centrifugation at 3,000 rpm for 2 min, followed by filtration through a 0.22-μm filter (Merck Millipore, Darmstadt, Germany). Ca^2+^ concentration was subsequently measured using inductively coupled plasma–optical emission spectrometry (ICP-OES; Agilent 5110, USA).

### RAW 264.7 cell culture

The RAW 264.7 murine macrophage cell line was obtained from Procell Life Science and Technology Co. Ltd. (CL-0190, Wuhan, China). Cells were cultured in complete medium containing Dulbecco’s modified Eagle’s medium (DMEM; C11885500BT, Thermo Fisher Scientific, Shanghai, China), supplemented with 10% (v/v) fetal bovine serum (FBS; ExCell Bio, Montevideo, Uruguay) and 1% (v/v) penicillin–streptomycin (Thermo Fisher Scientific, Shanghai, China). FBS was heat-inactivated by incubation at 56 °C for 30 min to eliminate complement activity prior to use. Cell cultures were maintained in a humidified incubator at 37 °C with 5% CO₂ atmosphere. Macrophages were passaged at a ratio of 1:3 upon achieving 80% to 90% confluence. For subsequent experiments, macrophages were seeded into 24-well plates at a density of 1 × 10^5^ cells per well.

### In vitro BHA-cell culture model

#### Establishment of the BHA direct culture model

BHA was prepared according to the protocol previously established by our research group and sterilized prior to use. BHA was weighed per well and evenly distributed to form a uniform layer approximately 1 or 2 mm in thickness. Macrophages were then seeded gently onto the BHA surface. During seeding, the pipette tip was placed against the sidewall of the well, and the cell suspension was added slowly to ensure even cell distribution and prevent particle displacement. Only wells with no visible gaps between BHA particles (confirmed by optical microscopy) were used for subsequent experiments.

#### Establishment of the BHA indirect culture model

BHA was incubated in the complete medium at the same material-to-liquid volume ratio as used in the direct culture model. After incubation at 37 °C for 24 h, the supernatant was collected, filtered through a 70-μm filter (Bioland, Shanghai, China), and centrifuged at 3,000 rpm for 2 min to obtain the BHA extract. The extract was immediately used for cell culture. After cells were seeded into the well for 6 h, the culture medium was removed and replaced with the extract.

#### RNA extraction

In situ lysis method: Following incubation with BHA, cells were lysed directly in the coculture system with lysis buffer.

Pre-separation method: The coculture system was washed twice with phosphate-buffered saline (PBS) to detach adherent cells from the materials. The wash solution was collected, filtered through a 70-μm cell strainer to remove material particles, and then centrifuged at 1,000 rpm for 5 min. The supernatant was completely removed, and the cell pellet was collected for further analysis. For RNA extraction, lysis buffer was added. Total RNA was extracted using the RNA-Quick Purification Kit (ESscience, Shanghai, China) according to the manufacturer’s protocol.

### Cell morphology observation

Following 24 h of culture, cells were fixed with 2.5% glutaraldehyde for 4 h at room temperature. Samples were then gently rinsed 5 times with PBS dehydrated through a graded ethanol series (30%, 50%, 70%, 90%, and 100% v/v) and immersed in tert-butanol for 15 min with 3 repetitions. Subsequently, samples were transferred to sequential freezing at −20 and −80 °C for 24 h, followed by lyophilization for 20 h using a freeze dryer (Christ, Osterode, Germany). Morphological characterization was performed via scanning electron microscopy (SEM; TESCAN MIRA LMS, Czech Republic).

### RT-qPCR

Following 6, 24, or 36 h of culture, macrophages were separated using the pre-separation method, and total RNA was extracted with the RNA-Quick Purification Kit. RNA concentration and purity were assessed via spectrophotometry (NanoDrop, Thermo Fisher Scientific, Waltham, USA) by measuring the absorbance ratios at 260/280 nm and 260/230 nm. cDNA was then synthesized with 500 ng of total RNA using a cDNA Synthesis SuperMix Kit (YEASEN, Shanghai, China). Reverse transcription quantitative polymerase chain reaction (RT-qPCR) was performed using qPCR SYBR Green Master Mix (11202ES08, YEASEN, Shanghai, China) on a QuantStudio 7 Real-Time PCR System (Applied Biosystems, Foster City, USA). The primer sequences of targeted genes (Table S1) were designed and verified using the National Center for Biotechnology Information (NCBI) databases. The expression of target genes was quantified via the 2^−ΔΔCT^ method, normalized to the internal control gene *Gapdh*.

### Enzyme-linked immunosorbent assay

To assess the secretion of inflammatory cytokines and chemokines, the cell culture supernatant was collected following 36 or 48 h of culture. Levels of tumor necrosis factor-α (TNF-α) and macrophage inflammatory protein-1α (MIP-1α) were quantified using enzyme-linked immunosorbent assay kits (CUSABIO, Wuhan, China) according to the manufacturer’s protocol.

### RNA-seq and bioinformatic analysis

Following 36 h of culture, macrophages were harvested and separated using the pre-separation method as mentioned in prior methodology. Total RNA was then extracted using the RNA-Quick Purification Kit according to the manufacturer’s protocol. RNA sequencing (RNA-seq) and bioinformatic analysis were carried out by BGI Genomics (Wuhan, China). Differentially expressed genes (DEGs) were defined as those with |fold change| >1.5 and adjusted *P* value < 0.05. Gene Ontology (GO), Kyoto Encyclopedia of Genes and Genomes (KEGG) enrichment analysis, and Gene Set Enrichment Analysis (GSEA) were performed to explore the gene functions and specific pathway activation.

### Intracellular Ca^2+^ detection

Intracellular Ca^2+^ concentration in macrophages was measured using a Fluo-4 Calcium Assay Kit (Beyotime Biotechnology, Shanghai, China). Following 36 h of culture, cell pellets were collected. Fluo-4 working solution was then prepared according to the manufacturer’s protocol and added to resuspend the cell pellets, and the mixture was incubated at 37 °C in the dark for 30 min. The cells were subsequently centrifuged at 1,000 rpm for 5 min to remove excess dye and resuspended in assay buffer for subsequent measurement. Intracellular calcium fluorescence intensity was measured using 2 methods: (a) Fluorescence microscopy: Cells were seeded into a 96-well black-walled, clear-bottom plate. Representative fluorescence images were captured using a fluorescence microscope (Axio, Zeiss, Germany) with green fluorescent protein (GFP) filter set (excitation: 470/40 nm, emission: 525/50 nm). (b) Flow cytometry: Analysis was conducted using a CytoFLEX flow cytometer (Beckman Coulter, Brea, CA, USA) equipped with a 488-nm blue laser. Fluorescence signals from Fluo-4 AM were detected through a 525/40-nm bandpass filter [fluorescein isothiocyanate (FITC) channel]. Data were acquired using CytExpert software (v2.4) and analyzed with FlowJo software (v10.10.0).

### TLR4-blockade experiment

Toll-like receptor 4 (TLR4) inhibitor TAK-242 (Resatorvid; MedChemExpress, USA) was dissolved in dimethyl sulfoxide (DMSO; MedChemExpress, USA) at a stock concentration of 200 μM and stored at −20 °C for subsequent experiments. For the BHA + TAK-242 group, macrophages were treated with DMEM containing 200 nM TAK-242 for 4 h. For the BHA group and control group, macrophages were treated with DMEM containing an equivalent volume of DMSO. The medium was removed after incubation, and the cells were harvested and seeded onto the BHA. For the BHA + TAK-242 group, a maintenance dose of TAK-242 (10% of the initial concentration) was added, while an equivalent volume of DMSO was added to the BHA and control groups to ensure consistency.

### Preparation and characterization of BHA(pre)

BHA was cyclically incubated in DMEM at a concentration of 10 mg/ml every 2 h for 3 or 6 cycles for calcium pre-adsorption, denoted as BHA(pre/3) or BHA(pre/6). BHA(pre) was immediately utilized for cell culture experiments.

To analyze the difference in calcium enrichment property between BHA and BHA(pre), the materials of different groups were incubated in complete medium at 37 °C for 24 h. The supernatant was collected by centrifugation at 3,000 rpm for 2 min, followed by filtration through a 0.22-μm filter. Ca^2+^ concentrations were subsequently tested using ICP-OES.

Zeta potential was measured using a solid-surface zeta potential analyzer (Anton Paar, Austria) in 1 mM KCl (pH 7.2). The surface morphology of BHA and BHA(pre) was observed via SEM (TESCAN MIRA LMS, Czech Republic). Elemental composition and spatial distribution were examined by energy-dispersive x-ray spectroscopy (EDS; Oxford Instruments, UK). The crystalline structure of different groups was analyzed by x-ray diffraction (XRD) (Rigaku SmartLab SE, Japan).

### Animal surgery and histological evaluation

Six-week-old male Sprague–Dawley rats were purchased from Guangdong Medical Laboratory Animal Center. All animal procedures were approved by the Institutional Animal Care and Use Committee of Sun Yat-sen University (approval no. SYSU-IACUC-2025-001708). For the subcutaneous implantation model, general anesthesia was induced using Zoletil 50 (Virbac, France). Tail vein blood was collected after warming the tail and disinfection with 75% ethanol. Whole blood was collected into coagulation-promoting tube and then centrifuged to obtain serum. Approximately 1 cm^2^ of each material (BHA, BHA + whole blood, or BHA + serum) was implanted into a subcutaneous pouch on the bilateral dorsum (*n* = 3 per group). Rats were euthanized 3 days after implantation. The implants with surrounding tissues were harvested and fixed in 4% paraformaldehyde (BL539A, Biosharp, China) for 24 h and then dehydrated in graded ethanol.

For histological analysis, specimens were decalcified in 4% EDTA, dehydrated, embedded in paraffin, and sectioned at 4-μm thickness, followed by deparaffinization and rehydration. Hematoxylin and eosin (H&E) staining was performed according to the manufacturer’s protocol. Macrophage infiltration was quantified in randomly selected regions of interest (ROIs; 1 mm^2^). Macrophages were identified based on morphological features and counted in 4 nonoverlapping high-power fields per section. Results were expressed as cell density (cells/mm^2^).

### Statistical analysis

All data are presented as mean ± standard deviation (SD). Homogeneity of variance was verified using Levene’s test before the statistical comparisons. For comparisons between 2 groups, the unpaired 2-tailed Student’s *t* test was applied. For comparisons of more than 2 groups, the one-way analysis of variance (ANOVA) with Tukey’s post hoc test was applied to assess intergroup differences. Statistical analysis was performed using GraphPad Prism 9.0.0, and *P* value < 0.05 was considered statistically significant (*P < 0.05, **P < 0.01, ***P < 0.001, ****P < 0.0001).

## Results and Discussion

### Two calcium-based microenvironments were distinguished for in vitro model construction

**Fig. 1.**
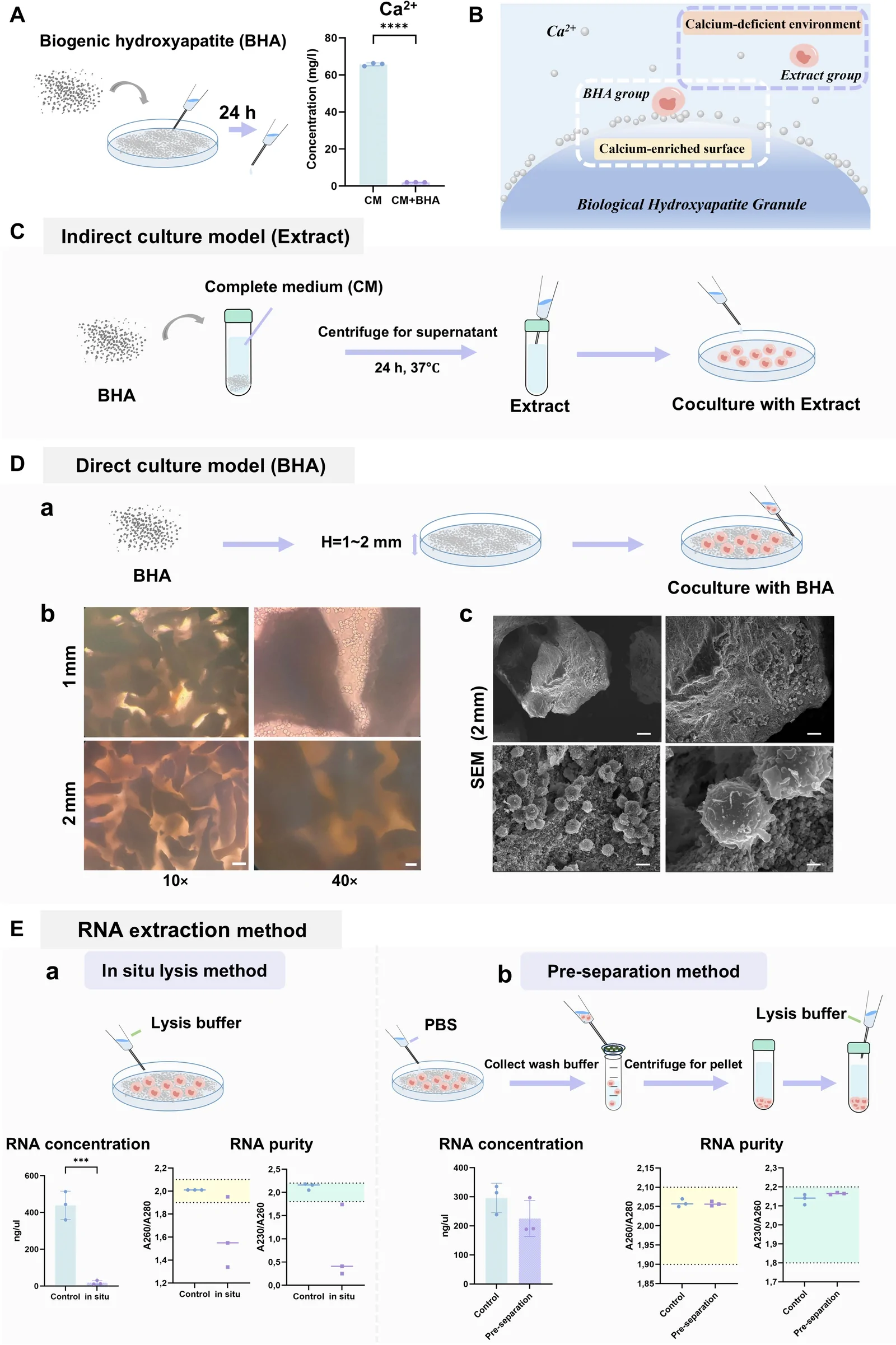
Establishment of culture models in vitro. (A) ICP-OES analysis of Ca^2+^ concentration in the supernatant after 24-h incubation of BHA in complete medium. (B) Schematic illustration of the 2 distinct microenvironments induced by BHA: the calcium-enriched hydroxyapatite surface and the surrounding calcium-deficient microenvironment. (C) Indirect culture model using BHA-conditioned medium. (D) (a) Direct culture model using BHA particles. (b) Representative optical microscopy images of the direct culture system with different BHA thicknesses (1 mm versus 2 mm). Magnification: 10× and 40×. (c) Representative SEM images of the 2-mm direct culture system. Scale bars, 50, 20, 5, and 1 μm. (E) Comparison of RNA extraction methods: (a) In situ lysis method: cells were lysed directly within the direct culture system. (b) Pre-separation method: cells were separated from the medium containing the materials by centrifugation prior to lysis.

Based on our standardized protocol, BHA derived from porcine cancellous bone was prepared [24–26]. ICP-OES analysis showed a significant decrease in Ca^2+^concentration in the supernatant after incubation with BHA (Fig. 1A), indicating a strong calcium enrichment capacity of hydroxyapatite, consistent with previous findings [27,28].

To determine whether cellular responses are driven by surface-associated interactions or the ion-conditioned environment, 2 distinct systems were established based on calcium distribution (Fig. 1B). A calcium-deficient environment was established using the indirect culture method with material extract as the stimulus (the Extract group; Fig. 1C), while a calcium-enriched BHA surface was established using the direct culture method (the BHA group; Fig. 1D).

For the direct culture model, a 1-mm BHA layer showed visible interparticle gaps under optical microscopy, with most cells attaching to the culture substrate rather than the material surface, indicating insufficient cell–material interaction. Increasing the thickness to 2 mm eliminated visible gaps and enabled homogeneous particle coverage [Fig. 1D(b)]. SEM further confirmed effective cell attachment on the BHA surface [Fig. 1D(c)].

For downstream molecular analysis, in situ lysis in the direct culture system yielded insufficient RNA quantity and quality [Fig. 1E(a)]. This limitation is consistent with the high surface adsorption capacity of hydroxyapatite for nucleic acids in particulate systems [29]. To address this, a pre-separation strategy was introduced to isolate cells prior to lysis, enabling recovery of RNA with sufficient yield [Fig. 1E(b)] and integrity (Fig. S1).

**Fig. 2.**
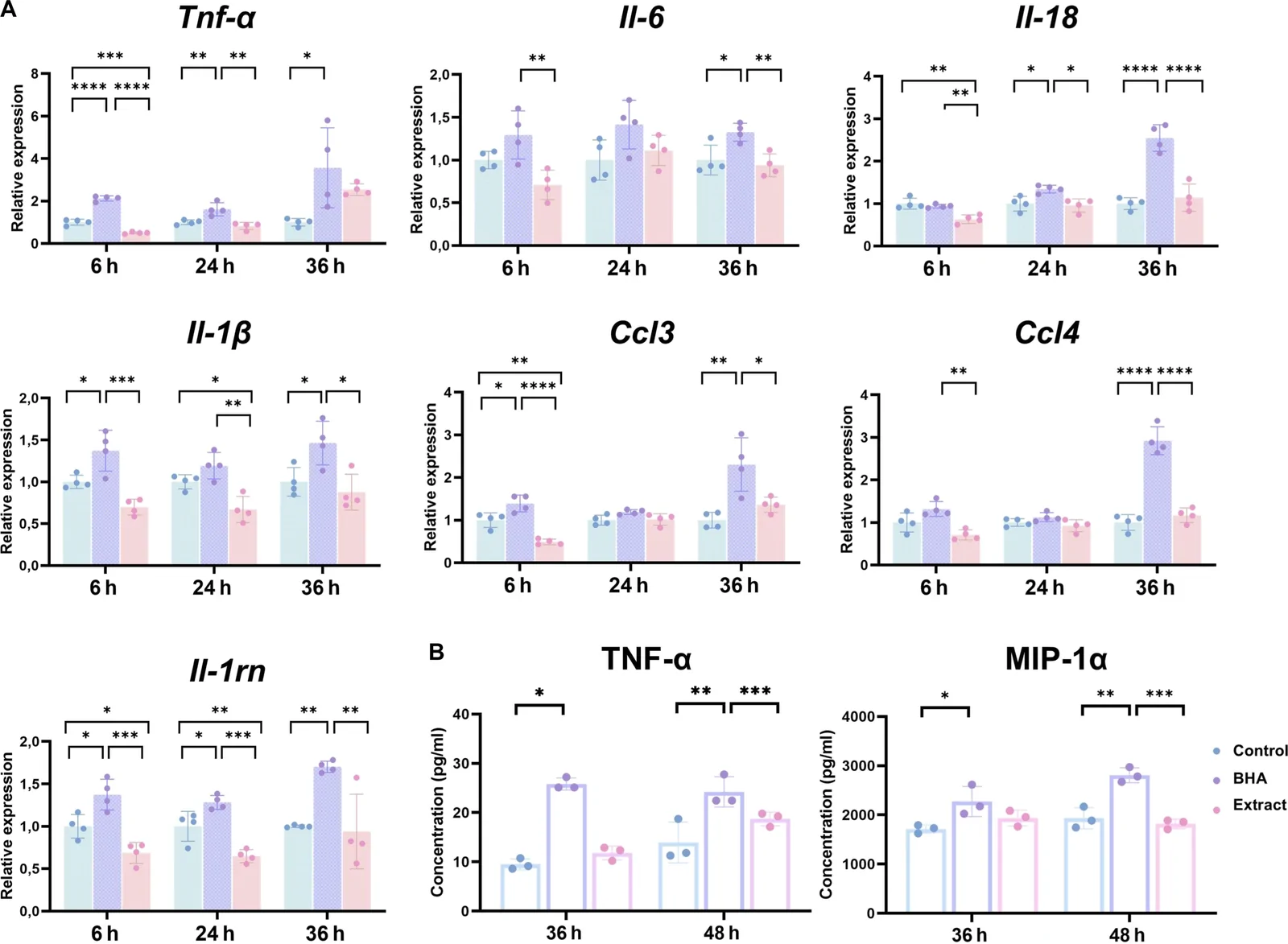
BHA induced a sequential immune response and inflammatory reaction in macrophages. (A) RT-qPCR analysis of inflammatory factor and chemokine expression in macrophages treated with BHA or Extract at 6, 24, and 36 h (*n* = 4). (B) ELISA analysis of TNF-α and MIP-1α levels in macrophages cultured with BHA or Extract at 36 and 48 h (*n* = 3). Data are presented as means ± SD. Statistical analysis was performed using the unpaired 2-tailed Student’s *t* test and one-way ANOVA followed by Tukey’s multiple comparison post hoc test (*P < 0.05, **P < 0.01, ***P < 0.001, ****P < 0.0001).

RT-qPCR was then performed to evaluate the inflammatory responses in direct culture models of different thicknesses. In the 1-mm system, *Tnf-α* and *Il-1rn* were significantly down-regulated compared with the control (Fig. S2A), whereas the 2-mm system induced up-regulation of *Tnf-α*, *Il-1rn*, and *Il-18* (Fig. S2B). These results indicate that sufficient contact is required to trigger inflammatory responses. Notably, the 1-mm system exhibited responses comparable to the Extract group in Fig. 2, suggesting that insufficient cell–material contact results in extract-dominant behavior.

Based on these findings, the 2-mm-thick direct culture model (the BHA group) was selected for subsequent experiments, with the Extract group retained as an ion-conditioned reference under identical material-to-medium ratios.

### The calcium-enriched surface of hydroxyapatite, rather than the calcium-deficient microenvironment, triggered inflammatory responses of macrophages

Macrophages play a central role in immune modulation and foreign body reactions, and are widely used to evaluate inflammatory responses induced by biomaterials [30,31]. Therefore, RAW 264.7 macrophages were selected for further investigation in our study. Cells were stimulated with BHA-conditioned medium (the Extract group) or directly cocultured with BHA particles (the BHA group), respectively.

RT-qPCR analysis of pro-inflammatory cytokines (*Tnf-α*, *Il-6*, *Il-18*, *Il-1β*, *Il-1rn*) and chemokines (*Ccl3*, *Ccl4*) showed a pronounced activation profile in the BHA group. Specifically, *Tnf-α*, *Il-18*, *Ccl3*, and *Ccl4* were sequentially up-regulated and peaked at 36 h. In contrast, the expression of these genes remained unchanged or down-regulated in the Extract group compared with the control (Fig. 2A). Consistently, enzyme-linked immunosorbent assay (ELISA) results demonstrated elevated TNF-α and MIP-1α levels only in the BHA group (Fig. 2B), indicating that inflammatory responses triggered by BHA particles are mediated by direct contact with the hydroxyapatite surface, whereas the surrounding calcium-deficient microenvironment does not induce inflammation.

**Fig. 3.**
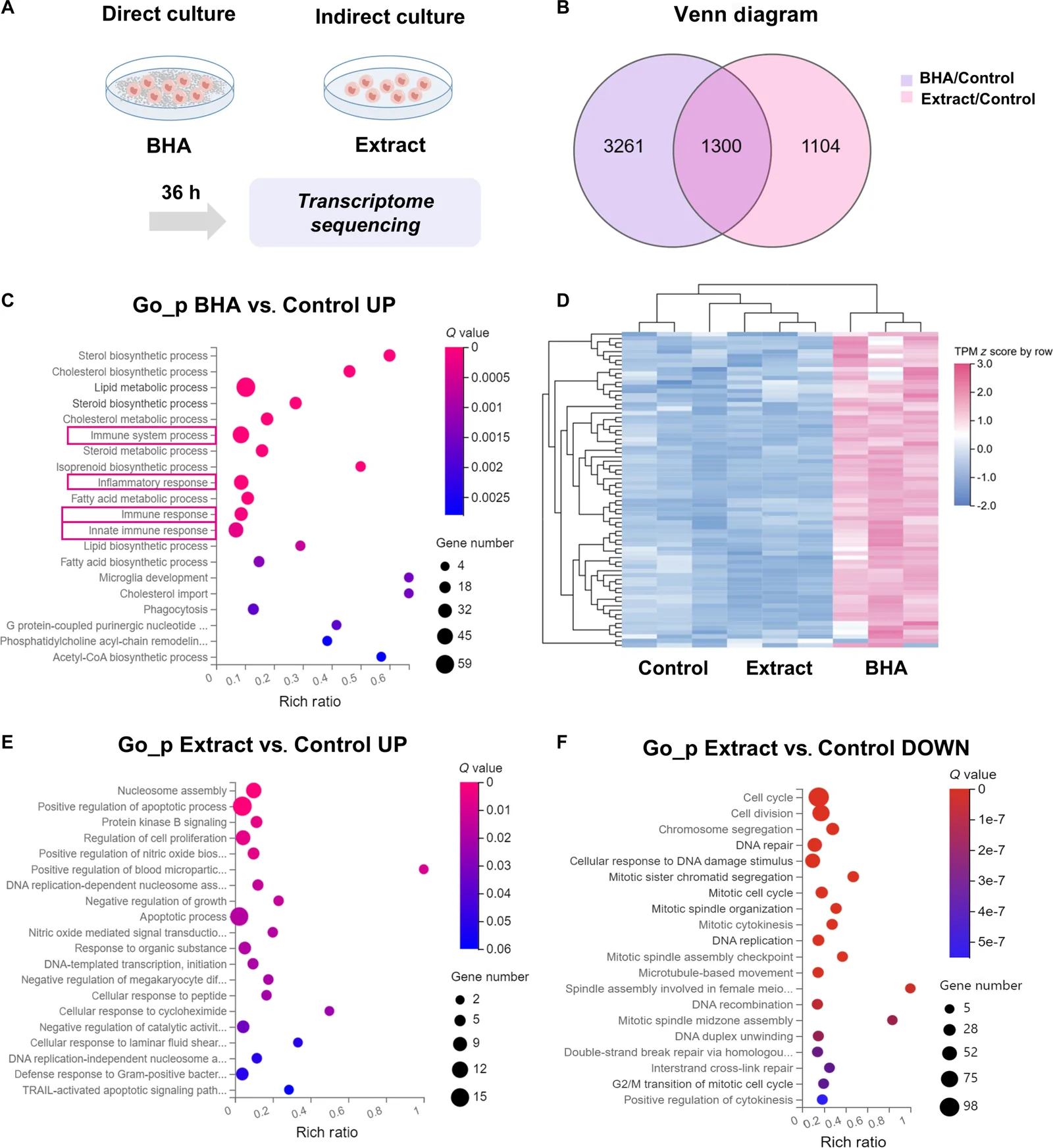
Hydroxyapatite surface, rather than the calcium-deficient environment, triggered inflammation in macrophages. (A) Schematic illustration: RNA was extracted from the direct or indirect culture systems at 36 h followed by transcriptome sequencing. (B) Venn diagram. (C) Top 20 terms of GO enrichment analysis for biological processes in up-regulated DEGs (BHA versus Control). (D) Heatmap of up-regulated DEGs for the terms “immune system process”, “immune response”, and “inflammatory response”. (E) Top 20 terms of GO enrichment analysis for biological processes in up-regulated DEGs (Extract versus Control). (F) Top 20 terms of GO enrichment analysis for biological processes in down-regulated DEGs (Extract versus Control). *DEGs are defined as those with |fold change| > 1.5 and adjusted *P* value < 0.05.

Given that gene expression peaked at 36 h, transcriptome analysis was performed at this time point to further clarify the underlying biological pathways. Principal components analysis (PCA) revealed reproducibility among samples (Fig. S3A). Variations in total gene expression were determined using Venn diagrams (Fig. 3B) and volcano plots (Fig. S3B). GO enrichment analysis revealed significant up-regulation of immune- and inflammation-related pathways in the BHA group (Fig. 3C and D), whereas such enrichment was absent in the Extract group (Fig. 3E). In contrast, the Extract group showed down-regulation of pathways associated with cell cycle, cell division, and DNA replication and repair (Fig. 3F), suggesting that a calcium-deficient microenvironment primarily affects cellular activity rather than triggering inflammatory activation, as reported in previous studies [32,33].

Overall, the results of transcriptome analysis were consistent with RT-qPCR and ELISA results, validating that BHA-induced inflammation is primarily driven by direct interaction with the calcium-enriched hydroxyapatite surface, rather than the calcium-deficient conditioned microenvironment.

### BHA induced TLR4 signaling and intracellular calcium elevation

It has been reported that nano-sized synthetic hydroxyapatite can induce inflammation via the TLR4 signaling pathway, leading to downstream activation of nuclear factor-κB (NF-κB) and interferon regulatory factor 3 (IRF3) [34,35]. Although clinically sized BHA differs in particle size, it shares a similar chemical composition. We therefore hypothesized that BHA may engage TLR4-related inflammatory signaling. Transcriptome analysis, RT-qPCR, and blockade experiments were performed to test this hypothesis.

**Fig. 4.**
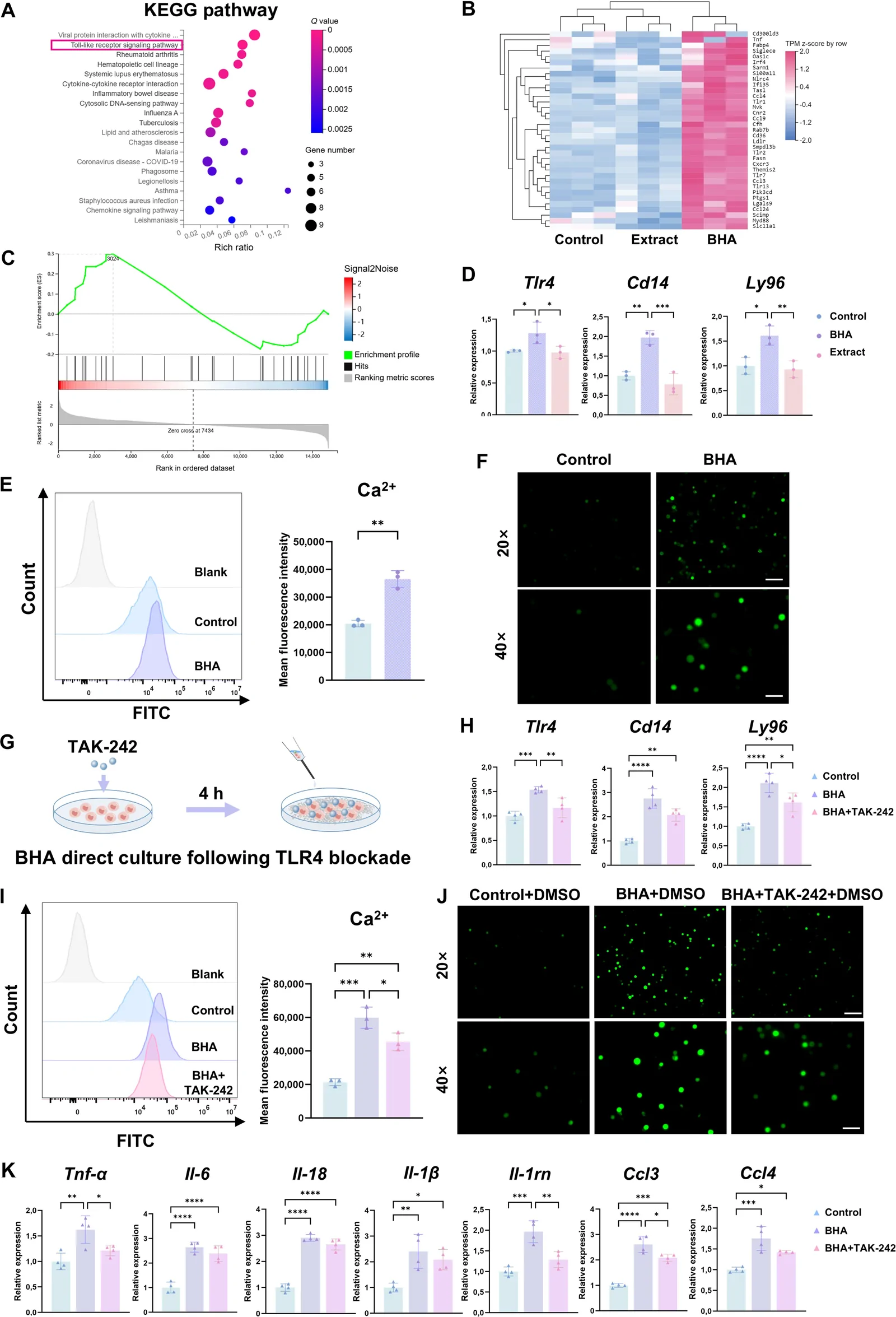
BHA activated the TLR4 pathway and elevated Ca^2+^ influx to trigger inflammatory responses in macrophages. (A) KEGG analysis of the top 20 pathways enriched in up-regulated DEGs (BHA versus Control). (B) Heatmap of up-regulated and down-regulated DEGs for the term “Toll-like receptor signaling pathway” (BHA versus Control). (C) GSEA analysis of the TLR4 signaling pathway. (D) RT-qPCR analysis of TLR4-related gene expression in the BHA, Extract, and Control groups at 36 h (*n* = 3). (E) Flow cytometry analysis of intracellular calcium levels by Fluo-4 AM staining (*n* = 3). (F) Representative fluorescence images of intracellular calcium in macrophages. Scale bars, 50 μm (20×) and 20 μm (40×). (G) For the BHA + TAK-242 group, macrophages were pretreated with TAK-242 and cocultured with BHA. (H) RT-qPCR analysis of TLR4-related gene expression (*n* = 4). (I) Flow cytometry analysis of intracellular calcium levels by Fluo-4 AM staining (*n* = 3). (J) Representative fluorescence images of intracellular calcium in macrophages. Scale bars, 50 μm (20×) and 20 μm (40×). (K) RT-qPCR analysis of inflammatory factor and chemokine expression in macrophages (*n* = 4). Data are presented as means ± SD. Statistical analysis was performed using the unpaired 2-tailed Student’s *t* test and one-way ANOVA followed by Tukey’s multiple comparison post hoc test (*P < 0.05, **P < 0.01, ***P < 0.001, ****P < 0.0001).

KEGG pathway analysis revealed significant enrichment of the “Toll-like receptor signaling pathway” in the BHA group compared with the control (Fig. 4A). The cluster heatmap showed that the control versus BHA comparison had the most up-regulated DEGs, whereas gene expression profiles were more similar between the Extract and control groups (Fig. 4B). Consistently, GSEA confirmed activation of the TLR4 signaling pathway in the BHA group (Fig. 4C).

RT-qPCR further showed significant up-regulation of TLR4-related gene (*Tlr4*, *Cd14*, and *Ly96*) at 36 h in the BHA group, while no significant changes were observed in the Extract group (Fig. 4D). These findings indicate that TLR4 activation is specifically triggered by direct interaction with the hydroxyapatite surface.

Given that TLR4 activation is often accompanied by calcium influx events in inflammatory responses [36–38], intracellular Ca^2+^ levels were further assessed. Flow cytometry and fluorescence imaging showed significantly increased intracellular Ca^2+^ in the BHA group (Fig. 4E and F), suggesting involvement of calcium influx in BHA-induced macrophage activation.

To further validate the role of TLR4, a blockade experiment was performed (Fig. 4G). RAW 264.7 cells were pretreated with TAK-242 prior to BHA exposure. RT-qPCR showed that TLR4 blockade significantly reduced the expression of *Tlr4*, *Cd14*, and *Ly96* and downstream genes *Tnf-α*, *Il-1rn*, *Ccl3*, and *Ccl4* (Fig. 4H and K). Meanwhile, intracellular Ca^2+^ levels were also significantly decreased in the BHA + TAK-242 group (Fig. 4I and J), suggesting that calcium influx is associated with TLR4-dependent macrophage activation.

In the BHA + TAK-242 group, TLR4-related gene expression remained higher than in the control group, suggesting the possible involvement of additional regulatory pathways beyond TLR4 signaling. In addition, *Il-6* and *Il-18* were not significantly down-regulated, which may be attributed to the complexity of inflammatory regulation and variations in stimulation conditions [39,40].

The surface mechanism underlying BHA-induced inflammation is closely associated with its calcium enrichment behavior. Kim et al. reported that the negatively charged hydroxyapatite surface forms a calcium-enriched layer via Ca^2+^ adsorption from the surrounding medium [27]. Our previous study further suggested that this early-stage calcium enrichment under charge-imbalanced conditions triggers inflammatory responses in macrophages [23]. Based on these findings, we hypothesized that modulation of calcium enrichment capacity alters surface electrostatic properties and thereby influences TLR4-related signaling.

**Fig. 5.**
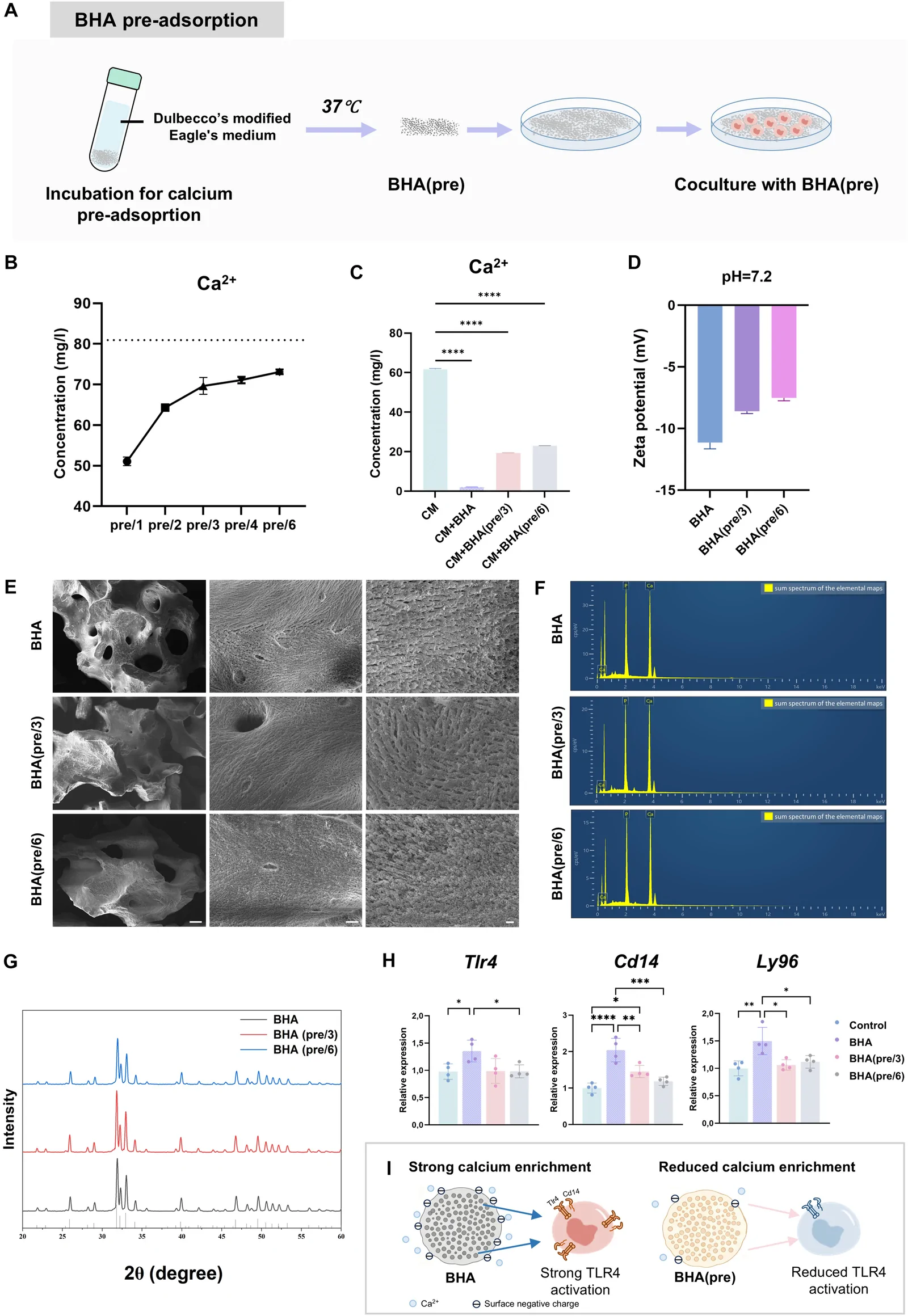
Surface charge modulation via calcium pre-adsorption attenuated TLR4 pathway activation in macrophages. (A) Schematic illustration: BHA was incubated in DMEM for multiple cycles for calcium pre-adsorption. (B and C) ICP-OES analysis of Ca^2+^ concentration in the supernatant after different pre-adsorption cycles (B) and after 24-h incubation in complete medium for BHA, BHA(pre/3), and BHA(pre/6) (C). (D) Zeta potential analysis of the different groups (pH 7.2). (E to G) Representative SEM images (scale bars, 100, 10, and 2 μm), EDS spectra, and XRD patterns of BHA, BHA(pre/3), and BHA(pre/6). (H) RT-qPCR analysis of TLR4-related gene expression in macrophages cultured with BHA, BHA(pre/3), or BHA(pre/6) (*n* = 4). (I) Schematic illustration of the proposed mechanism: Calcium pre-adsorption reduces surface electronegativity and calcium enrichment activity, thereby attenuating TLR4 activation in macrophages.

To test this, a calcium pre-adsorption strategy was employed. BHA was pre-adsorbed with DMEM for different cycles. ICP-OES analysis showed a cycle-dependent increase in residual Ca^2+^ concentration in the supernatant, reflecting reduced calcium enrichment capacity of BHA(pre). A plateau was observed after 3 to 6 cycles; accordingly, BHA(pre/3) and BHA(pre/6) were defined. Further analysis confirmed a progressive decrease in calcium adsorption compared with untreated BHA (Fig. 5B and C).

Zeta potential analysis showed that the untreated BHA surface was negatively charged (−11.14 mV). With increasing pre-adsorption cycles, the surface potential gradually shifted to −8.60 mV and −7.51 mV in BHA(pre/3) and BHA(pre/6), respectively. SEM, XRD, and EDS analyses confirmed no detectable changes in morphology, crystallinity, or elemental composition (Fig. 5D to G). RT-qPCR results further showed that calcium pre-adsorption down-regulated TLR4-related gene expression, indicating suppression of TLR4 activation (Fig. 5H). This trend is consistent with previous studies showing that changes in the surface electrostatic properties of lipopolysaccharide aggregates can influence TLR4 activation [41].

Collectively, these results suggest that calcium pre-adsorption attenuates BHA-induced inflammatory responses by reducing surface calcium enrichment and modulating surface electrostatic properties, thereby suppressing TLR4 activation in macrophages.

### Calcium pre-adsorption attenuated BHA-induced inflammatory responses via modulation of TLR4 activation

**Fig. 6.**
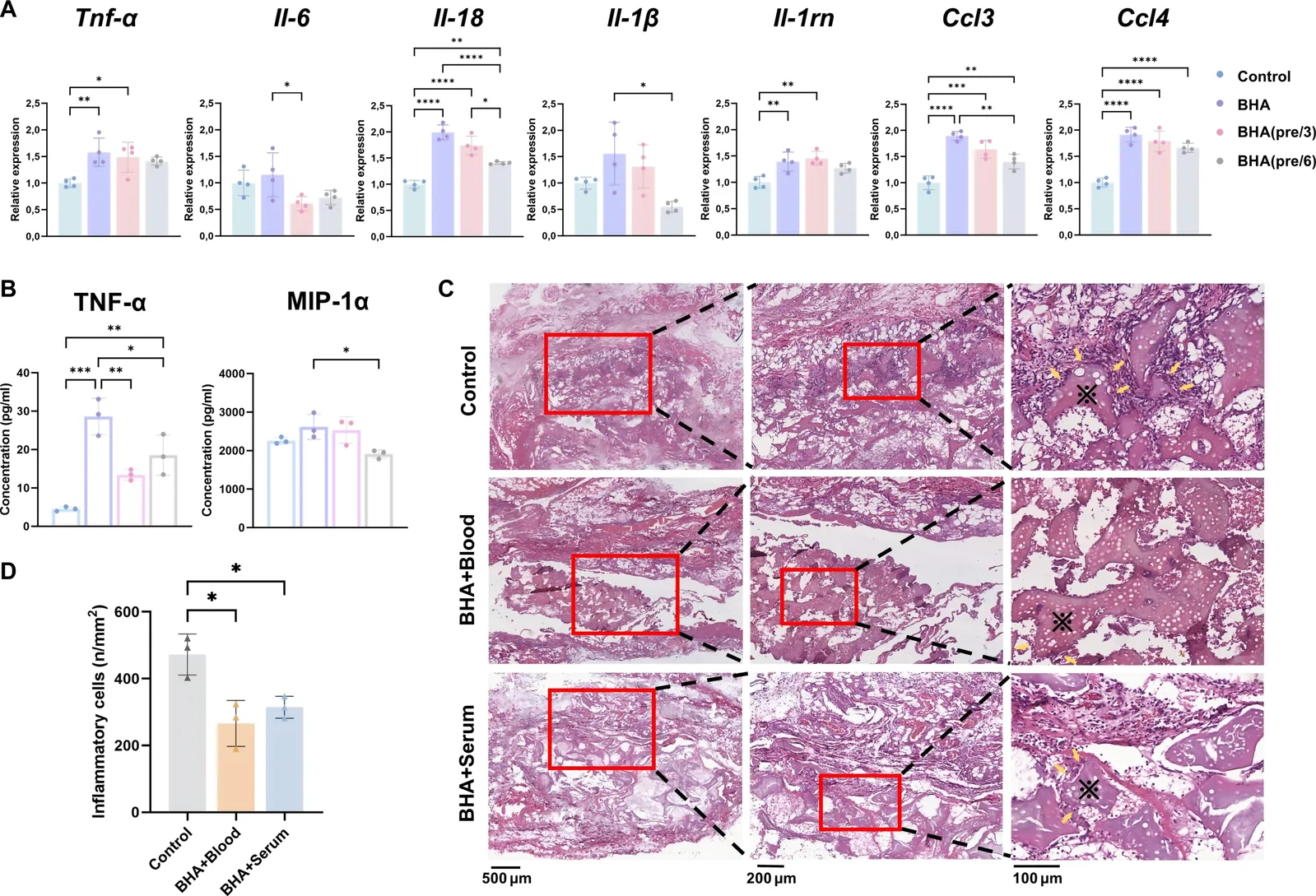
Calcium pre-adsorption attenuated inflammatory responses in vitro and early macrophage infiltration induced by BHA implantation. (A) RT-qPCR analysis of inflammatory factor and chemokine expression in macrophages cultured with BHA, BHA(pre/3), or BHA(pre/6) (*n* = 4). (B) ELISA analysis of TNF-α and MIP-1α levels in macrophages cultured with BHA, BHA(pre/3), or BHA(pre/6) (*n* = 3). (C) Representative H&E staining images of the BHA, BHA + Blood, and BHA + Serum groups. Yellow arrows indicate inflammatory cells. (D) Quantification of inflammatory cell density. Data are presented as means ± SD. Statistical analysis was performed using the unpaired 2-tailed Student’s *t* test and one-way ANOVA followed by Tukey’s multiple comparison post hoc test (*P < 0.05, **P < 0.01, ***P < 0.001, ****P < 0.0001).

Having demonstrated that calcium pre-adsorption reduced surface electronegativity while preserving the structure of BHA, we next evaluated its effect on the downstream inflammatory responses. RT-qPCR results showed that calcium pre-adsorption significantly down-regulated the expression of key inflammatory genes compared with the untreated BHA. Among these, only Il-18 exhibited a further reduction in the BHA(pre/6) group relative to the BHA(pre/3) group (Fig. 6A). Consistently, ELISA results showed decreased TNF-α levels in both BHA(pre/3) and BHA(pre/6) groups, while MIP-1α was significantly reduced only in the BHA(pre/6) group (Fig. 6B), indicating a cycle-dependent reduction in inflammatory activity.

Pre-adsorption was conducted in serum-free DMEM to avoid interference from serum proteins, whereas subsequent cell culture was performed in complete medium containing 10% FBS. Although ion exchange may still occur under culture conditions, the reduced surface electronegativity of BHA(pre) is expected to maintain a lower calcium enrichment level than untreated BHA, resulting in attenuated inflammation.

To assess translational relevance, a preliminary in vivo subcutaneous implantation model was established using clinically relevant pre-adsorption media. BHA was pretreated with whole blood (BHA + Blood) or serum (BHA + Serum) prior to the implantation. Histological analysis at day 3 revealed marked macrophage infiltration around untreated BHA particles, whereas both the BHA + Blood and the BHA + Serum groups exhibited significantly reduced inflammatory cell accumulation, with the most pronounced reduction observed in the BHA + Blood group (Fig. 6C and D).

Collectively, these findings demonstrate that calcium pre-adsorption attenuated BHA-induced inflammatory responses in macrophages, which is consistent with its role in modulating surface electrostatic properties and TLR4 signaling. This strategy preserved the structural integrity of hydroxyapatite while providing a practical approach to regulate the early inflammatory responses. However, the long-term stability of the pre-adsorbed surface under physiological conditions and its impact on osteogenesis remain to be investigated. Optimization of pre-adsorption media, treatment cycles, and clinically relevant protocols will be necessary for future translational applications. Further in vivo studies with extended observation are required to validate its translational potential.

## Conclusion

A novel in vitro direct culture model and RNA extraction strategy were established for granular particles, enabling complete cell–material contact and yielding sufficient high-quality RNA for downstream analysis. BHA activated inflammation in macrophages via the TLR4–calcium signaling pathway, primarily driven by direct contact with the calcium-enriched surface rather than the surrounding calcium-deficient environment. Calcium pre-adsorption effectively reduced the surface calcium enrichment activity while preserving the microscopic morphology of BHA, attenuating the downstream inflammatory response. These findings elucidate the pivotal role of surface calcium enrichment in hydroxyapatite-induced inflammation and provide a potential modulation strategy for developing novel low-inflammatory bone substitutes.

## Data Availability

Data will be made available on request.

## References

[1] Deng Y, Liang Y, Liu X. Biomaterials for periodontal regeneration. Dent Clin N Am. 2022;66(4):659–672. 36216452 10.1016/j.cden.2022.05.011 PMC10014143

[2] Nishida J, Shiraishi H, Okada K, Ehara S, Shimamura T. Vascularized iliac bone graft for iliosacral bone defect after tumor excision. Clin Orthop Relat Res. 2006;447:145–151. 16505711 10.1097/01.blo.0000203485.90711.1b

[3] Wang J, Liu M, Yang C, Pan Y, Ji S, Han N, Sun G. Biomaterials for bone defect repair: Types, mechanisms and effects. Int J Artif Organs. 2024;47(2):75–84. 38166512 10.1177/03913988231218884

[4] Cypher TJ, Grossman JP. Biological principles of bone graft healing. J Foot Ankle Surg. 1996;35(5):413–417. 8915864 10.1016/s1067-2516(96)80061-5

[5] Zhao R, Yang R, Cooper PR, Khurshid Z, Shavandi A, Ratnayake J. Bone grafts and substitutes in dentistry: A review of current trends and developments. Molecules. 2021;26(10):3007. 34070157 10.3390/molecules26103007 PMC8158510

[6] Schmitt CM, Doering H, Schmidt T, Lutz R, Neukam FW, Schlegel KA. Histological results after maxillary sinus augmentation with Straumann^®^ BoneCeramic, Bio-Oss^®^, Puros^®^, and autologous bone: A randomized controlled clinical trial. Clin Oral Implants Res. 2013;24(5):576–585. 22324456 10.1111/j.1600-0501.2012.02431.x

[7] Velard F, Braux J, Amedee J, Laquerriere P. Inflammatory cell response to calcium phosphate biomaterial particles: An overview. Acta Biomater. 2013;9(2):4956–4963. 23036944 10.1016/j.actbio.2012.09.035

[8] Miron RJ, Bohner M, Zhang Y, Bosshardt DD. Osteoinduction and osteoimmunology: Emerging concepts. Periodontol 2000. 2024;94(1):9–26. 37658591 10.1111/prd.12519

[9] Negrescu AM, Cimpean A. The state of the art and prospects for osteoimmunomodulatory biomaterials. Materials. 2021;14(6):1357. 33799681 10.3390/ma14061357 PMC7999637

[10] Yang N, Liu Y. The role of the immune microenvironment in bone regeneration. Int J Med Sci. 2021;18(16):3697–3707. 34790042 10.7150/ijms.61080 PMC8579305

[11] Chen Z, Yuen J, Crawford R, Chang J, Wu C, Xiao Y. The effect of osteoimmunomodulation on the osteogenic effects of cobalt-incorporated β-tricalcium phosphate. Biomaterials. 2015;61:126–138. 26001077 10.1016/j.biomaterials.2015.04.044

[12] Zhou X, Xue J, Zhang Y, Xia R, Shan Z, Zhang L, Gui M, Liu G, Chen Z. Evolution of biological hydroxyapatite modification strategy: Anti-inflammation approach rescues the calcium–NOD-like receptor–inflammation axis-mediated periodontal redevelopment failure. Biomater Res. 2025;29:131. 10.34133/bmr.0131 PMC11862812 40012607

[13] Chen K, Ha S, Xu L, Liu C, Liu Y, Wu X, Li Z, Wu S, Yang B, Chen Z. Fluorinated hydroxyapatite conditions a favorable osteo-immune microenvironment via triggering metabolic shift from glycolysis to oxidative phosphorylation. J Transl Med. 2024;22(1):437. 38720345 10.1186/s12967-024-05261-0 PMC11077739

[14] Kofina V, Monfaredzadeh M, Rawal SY, Dentino AR, Singh M, Tatakis DN. Patient-reported outcomes following guided bone regeneration: Correlation with clinical parameters. J Dent. 2023;136: Article 104605. 37419383 10.1016/j.jdent.2023.104605

[15] Zazou N, Diab N, Bahaa S, El Arab AE, Aziz OA, El Nahass H. Clinical comparison of different flap advancement techniques to periosteal releasing incision in guided bone regeneration: A randomized controlled trial. Clin Implant Dent Relat Res. 2021;23(1):107–116. 33155422 10.1111/cid.12960

[16] Kofina V, Monfaredzadeh M, Rawal SY, Dentino AR, Singh M, Tatakis DN. Guided bone regeneration-associated tissue swelling: A digital three-dimensional assessment. J Prosthet Dent. 2025;134(6):2359–2367. 39366838 10.1016/j.prosdent.2024.09.009

[17] Krattiger LA, Guex AG. Complex in vitro model systems to understand the biointerfaces of dental implants. Dent Mater. 2025;41(7):810–826. 40360331 10.1016/j.dental.2025.05.001

[18] Mu Y, Du Z, Gao W, Xiao L, Crawford R, Xiao Y. The effect of a bionic bone ionic environment on osteogenesis, osteoimmunology, and in situ bone tissue engineering. Biomaterials. 2024;304: Article 122410. 38043465 10.1016/j.biomaterials.2023.122410

[19] Yao X, Peng R, Ding J. Cell-material interactions revealed via material techniques of surface patterning. Adv Mater. 2013;25(37):5257–5286. 24038153 10.1002/adma.201301762

[20] Nguyen AT, Sathe SR, Yim EKF. From nano to micro: Topographical scale and its impact on cell adhesion, morphology and contact guidance. J Phys Condens Matter. 2016;28(18): Article 183001. 27066850 10.1088/0953-8984/28/18/183001

[21] Sadowska JM, Guillem-Marti J, Espanol M, Stähli C, Döbelin N, Ginebra MP. In vitro response of mesenchymal stem cells to biomimetic hydroxyapatite substrates: A new strategy to assess the effect of ion exchange. Acta Biomater. 2018;76:319–332. 29933107 10.1016/j.actbio.2018.06.025

[22] Mitran V, Ion R, Miculescu F, Necula MG, Mocanu AC, Stan GE, Antoniac IV, Cimpean A. Osteoblast cell response to naturally derived calcium phosphate-based materials. Materials. 2018;11(7):1097. 29954120 10.3390/ma11071097 PMC6073128

[23] Li C, Su M, Mai M, Guo Z, Li Y, Chen S, Liu Q, Chen D, Wu X, Chen Z, et al. Calcium enrichment activity initiates extracellular calcium influx-dependent inflammatory response of biologically derived hydroxyapatite. Mater Today Bio. 2024;28: Article 101231. 10.1016/j.mtbio.2024.101231 PMC11409067 39296358

[24] Liu Q, Chen Z, Gu H, Chen Z. Preparation and characterization of fluorinated porcine hydroxyapatite. Dent Mater J. 2012;31(5):742–750. 23037836 10.4012/dmj.2012-052

[25] Qiao W, Liu Q, Li Z, Zhang H, Chen Z. Changes in physicochemical and biological properties of porcine bone-derived hydroxyapatite induced by fluoride incorporation. Sci Technol Adv Mater. 2017;18(1):110–121. 28243337 10.1080/14686996.2016.1263140 PMC5315024

[26] Liu R, Qiao W, Huang B, Chen Z, Fang J, Li Z, Chen Z. Fluorination enhances the osteogenic capacity of porcine hydroxyapatite. Tissue Eng Part A. 2018;24(15–16):1207–1217. 29376480 10.1089/ten.TEA.2017.0381

[27] Kim HM, Himeno T, Kokubo T, Nakamura T. Process and kinetics of bonelike apatite formation on sintered hydroxyapatite in simulated body fluid. Biomaterials. 2005;26(21):4366–4373. 15701365 10.1016/j.biomaterials.2004.11.022

[28] Sadowska JM, Guillem-Marti J, Ginebra MP. Influence of physicochemical properties of biomimetic hydroxyapatite on endothelial progenitor cells. Adv Healthc Mater. 2019;8(2): Article e1801138. 30516356 10.1002/adhm.201801138

[29] Liu X, Yin H, Liu H, Cai Y, Qi X, Dang Z. Multicomponent adsorption of heavy metals onto biogenic hydroxyapatite: Surface functional groups and inorganic mineral effects. J Hazard Mater. 2023;443(Pt A): Article 130167. 36270188 10.1016/j.jhazmat.2022.130167

[30] Anderson JM, Rodriguez A, Chang DT. Foreign body reaction to biomaterials. Semin Immunol. 2008;20(2):86–100. 18162407 10.1016/j.smim.2007.11.004 PMC2327202

[31] Su M, Li C, Deng S, Xu L, Shan Z, Xing Y, Li X, Li Y, Liu X, Zhong X, et al. Balance between the CMC/ACP nanocomplex and blood assimilation orchestrates immunomodulation of biomineralized collagen matrix. ACS Appl Mater Interfaces. 2023;15(50):58166–58180. 38079631 10.1021/acsami.3c12390

[32] Wagner AS, Glenske K, Henß A, Kruppke B, Rößler S, Hanke T, Moritz A, Rohnke M, Kressin M, Arnhold S, et al. Cell behavior of human mesenchymal stromal cells in response to silica/collagen-based xerogels and calcium-deficient culture conditions. Biomed Mater. 2017;12(4): Article 045003. 28425919 10.1088/1748-605X/aa6e29

[33] Schreiber R. Ca^2+^ signaling, intracellular pH and cell volume in cell proliferation. J Membr Biol. 2005;205(3):129–137. 16362501 10.1007/s00232-005-0778-z

[34] Hua Y, Wu J, Wu H, Su C, Li X, Ao Q, Zeng Q, Zhu X, Zhang X. Exposure to hydroxyapatite nanoparticles enhances Toll-like receptor 4 signal transduction and overcomes endotoxin tolerance in vitro and in vivo. Acta Biomater. 2021;135:650–662. 34525415 10.1016/j.actbio.2021.09.006

[35] Wang R, Hua Y, Wu H, Wang J, Xiao YC, Chen X, Ao Q, Zeng Q, Zhu X, Zhang X. Hydroxyapatite nanoparticles promote TLR4 agonist-mediated anti-tumor immunity through synergistically enhanced macrophage polarization. Acta Biomater. 2023;164:626–640. 37086827 10.1016/j.actbio.2023.04.027

[36] Geng J, Shi Y, Zhang J, Yang B, Wang P, Yuan W, Zhao H, Li J, Qin F, Hong L, et al. TLR4 signalling via Piezo1 engages macrophage host response during bacterial infection. Nat Commun. 2021;12(1):3519. 34112781 10.1038/s41467-021-23683-y PMC8192512

[37] Sun R, Zhu Z, Su Q, Li T, Song Q. Toll-like receptor 4 is involved in bacterial endotoxin-induced endothelial injury and SOC-mediated calcium regulation. Cell Biol Int. 2012;36(5):475–481. 22288713 10.1042/CBI20110535

[38] Schappe MS, Szteyn K, Stremska ME, Mendu SK, Downs TK, Seegren PV, Mahoney MA, Dixit S, Krupa JK, Stipes EJ, et al. TRPM7 mediates Ca2+ influx essential for LPS-induced TLR4 endocytosis and macrophage activation. Immunity. 2018;48(1):59–74.e5. 29343440 10.1016/j.immuni.2017.11.026 PMC5783319

[39] Yang X, Tao X, Qi W, Liu Z, Wang Y, Han Q, Xu C. TLR4 targeting contributes to recovery of osteoimmunology in periodontitis. J Periodontal Res. 2021;56(4):782–788. 33729573 10.1111/jre.12877

[40] Ono Y, Maejima Y, Saito M, Sakamoto K, Horita S, Shimomura K, Inoue S, Kotani J. TAK-242, a specific inhibitor of TLR4 signalling, prevents endotoxemia-induced skeletal muscle wasting in mice. Sci Rep. 2020;10(1):694. 31959927 10.1038/s41598-020-57714-3 PMC6970997

[41] Zamyatina A, Heine H. Lipopolysaccharide recognition in the crossroads of TLR4 and caspase-4/11 mediated inflammatory pathways. Front Immunol. 2020;11: Article 585146. 33329561 10.3389/fimmu.2020.585146 PMC7732686

