# Supplementary material for: Surface Calcium Enrichment of Biogenic Hydroxyapatite Regulates Macrophage Inflammation via Toll-Like Receptor 4 Signaling
Source: Biomater Res. 2026 Sep 1;30:0409. doi: 10.34133/bmr.0409 (PMC13530371; doi:10.34133/bmr.0409)
Supplement: Supplementary file 1 [file bmr.0409.f1.docx]

**Supplementary Materials**

**Figure S1. RNA quality control gel image.** RNA was extracted using the pre-separation method from the Control (1-3), BHA (4-6) and Extract (7-9) groups. M1 represents the standard DNA marker.

**Figure S2. Inflammatory gene expression in direct culture models of different BHA thicknesses.** (A) RT-qPCR analysis of inflammatory gene expression in macrophages using the direct culture model with a 1-mm-thick BHA layer. (B) RT-qPCR analysis of inflammatory gene expression in macrophages using the direct culture model with a 2-mm-thick BHA layer.

**Figure S3.** (A) Principal component analysis (PCA) of the Control, BHA and Extract groups (n = 3). (B,C) Volcano plots of differentially expressed genes (DEGs) in RNA sequencing analysis (BHA vs. Control, Extract vs. Control).

**Table S1.** Primer sequences.

| **Genes** | **Prim**er Sequences (from 5’ to 3’) |
| --- | --- |
| *Tnf-α* | Forward: GGATCTCAAAGACAACCAAC |
|  | Reverse: ACAGAGCAATGACTCCAAAG |
| *Il-6* | Forward: GATAAGCTGGAGTCACAGAAGG |
|  | Reverse: GGAATGTCCACAAACTGATATGC |
| *Il-18* | Forward: TGGCCGACTTCACTGTACAAC |
|  | Reverse: TGGGGTTCACTGGCACTTTG |
| *Il-1β* | Forward: TGCCACCTTTTGACAGTGATG |
|  | Reverse: ATGTGCTGCTGCGAGATTTG |
| *Il-1rn* | Forward: ACCAAATATCAAACTAGAAGAAAA |
|  | Reverse: CAGAGCGGATGAAGGTAA |
| *Ccl3* | Forward: ACACTCTGCAACCAAGTCTTC |
|  | Reverse: AGGAAAATGACACCTGGCTG |
| *Ccl4* | Forward: ATGAAGCTCTGCGTGTCTGC |
|  | Reverse: GCCTCTTTTGGTCAGGAATACCAC |
| *Tlr4* | Forward: TCCCTGCATAGAGGTAGTTCC  Reverse: TCAAGGGGTTGAAGCTCAGA |
| *Cd14* | Forward: TTTCAGAATCTACCGACCATGGAGC  Reverse: ACAATTCCACATCTGCCGCC |
| *Ly96*  *Gapdh* | Forward: CAACAGTGGTTCTGCAACTC  Reverse: CTCTTGGAATGAACTCAACATGC |
|  | Forward: TGACCACAGTCCATGCCATC |
|  | Reverse: GACGGACACATTGGGGGTAG |
